# The inner tube effect

**DOI:** 10.1177/20416695241249129

**Published:** 2024-05-08

**Authors:** Ian M. Thornton, Sunčica Zdravković, Dejan Todorović

**Affiliations:** Department of Cognitive Science, Faculty of Media and Knowledge Sciences, University of Malta, Msida, Malta; Laboratory of Experimental Psychology, Department of Psychology, Faculty of Philosophy, University of Novi Sad, Novi Sad, Serbia; Laboratory of Experimental Psychology, University of Belgrade, Belgrade, Serbia; Laboratory of Experimental Psychology, University of Belgrade, Belgrade, Serbia

**Keywords:** visual illusions, rocking line illusion, RLI, size illusions, contrast illusions

## Abstract

We describe a novel size illusion in which targets appear to either shrink or grow when enclosed within a narrow tube. The direction of size change is determined by the contrast step between display elements. We first noticed this effect in the context of the dynamic “rocking line” illusion (RLI), but it can also be easily seen in completely static displays. As with the RLI, the overall scale of the display seems to play an important role. We provide an online, interactive demo, enabling the reader to explore the relevant parameter space.

Recently, we described a novel dynamic illusion in which a smoothly translating object is seen to “rock” around its own center (Thornton & Todorović, 2023). The “rocking line” illusion (RLI) uses a very simple display that consists of two components: a rectangular horizontal target object and a background context where horizontally staggered rectangles of similar size to the target form a narrow checkboard pattern (Figure 1). When the display is appropriately scaled – with all objects subtending < 1.0° visual angle – a compelling impression of rocking occurs. Specifically, the target appears to deviate from its veridical horizontal path and instead appears to “rock” as it moves.

**Figure 1.**
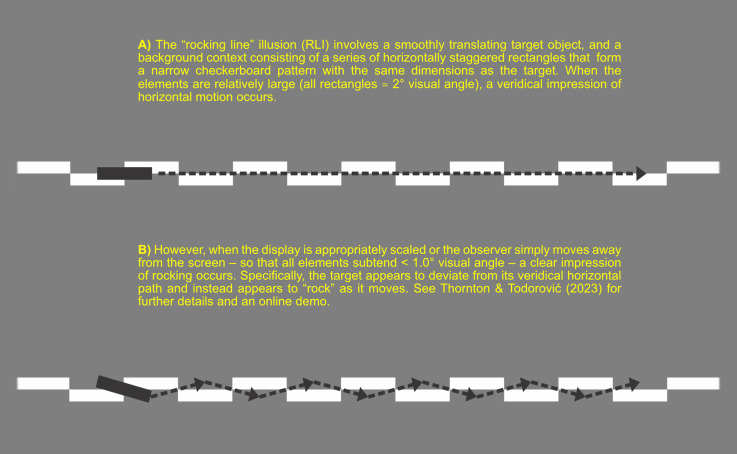
Schematic illustration of the rocking line illusion (Thornton & Todorović, 2023).

Our original explanation for the RLI – based on the model and simulations in Todorović (2021) – focused on the role of contrast polarity gradients that are formed each time the target object moves across an X-junction in the background (Thornton & Todorović, 2023). The purpose of the current short report is to describe an additional factor that we now think also plays an important role in the RLI, a factor that is itself quite compelling. The effect in question – which we are calling the “inner tube effect” (ITE) – can be seen in Movie 1.

**Movie 1.** A circular version of the RLI, with two illusory targets and two control targets. The two control targets move smoothly and appear fatter than the two illusory targets. Notice that fixation attenuates the rocking impression but not the size effect. https://maltacogsci.org/ITE/ITE_Movie1.html

Movie 1 shows a circular version of the RLI. Two of the black rectangles – the illusory targets –appear to rock quite strongly as they move, just as in the original linear version. Moving back from the screen should enhance this effect. Notice that the two rocking targets also appear to be thinner than the two other (control) rectangles. This is the “inner tube effect” (ITE).

In reality all four rectangles are identical and are moving in a smooth trajectory, with their paths placed directly on the midline of the white staggered background. The reader can confirm this by visiting the online demo at https://maltacogsci.org/ITE/ where the background ring can be toggled on and off, as well as other parameters adjusted. See also the OSF page associated with this paper at https://osf.io/9cpfd/.

The two control rectangles appear different because they are surrounded by background grey “wings” that erase the flanking white elements in the direction orthogonal to motion. This reveals that the white flanking elements give rise to both a motion illusion (RLI) and a size illusion (ITE). The apparent thinning of the two control items – the ITE – is the focus of the current paper.

The first point to note is that the ITE is independent of the RLI. This is illustrated in Movie 2, where we have replaced the staggered background of the RLI with a continuous “tube”. The four target items are identical to those in Movie 1.

**Figure 2.**
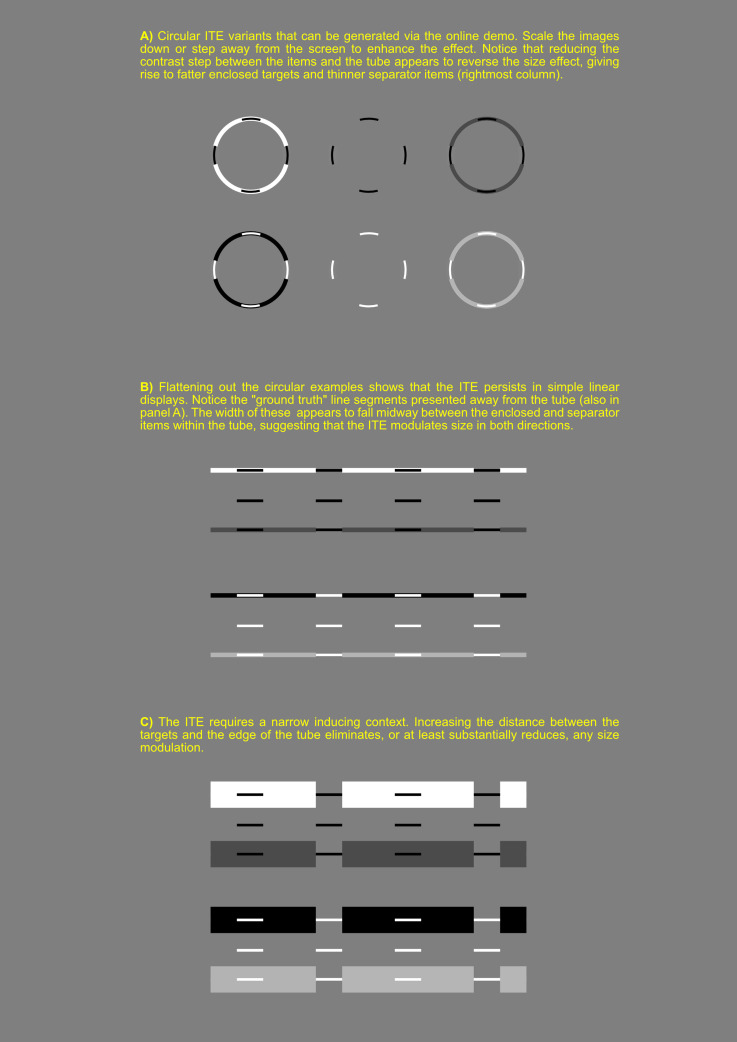
The inner tube effect (ITE) illustrated in static displays.

**Movie 2.** The inner tube effect (ITE). The four black segments are identical to each other. The two targets on the white background – within the tube – appear compressed in size. https://maltacogsci.org/ITE/ITE_Movie2.html

In Movie 2, the contrast in apparent size between the two targets fully flanked by the white tube, and the two separator items between the semi-circles, with grey surround, seems even stronger. Again, the online demo can be used to verify that all four black objects are identical.

The second point to note is that the ITE does not rely on motion. This will become clear if you pause Movie 2, but is also illustrated in Figure 2, which presents a range of static configurations.

So, how does the ITE contribute to the RLI? The RLI relies on minimized “checkerboard” patterns in which the target object moves directly along the midline of staggered background elements. At any one moment, the target will have a white flanking border on one side, and a grey flanking border on the other (see Figure 1, panel A). The white border itself has a clearly defined edge with the background, giving rise to a narrow “tube” on one side. As noted above, the white flanking “tube” is likely to compress the apparent size of the target on the edge with which it overlaps. This may give the impression that the object shrinks on the white side, and/or grows to fill the blank, grey space opposite.

In the linear version of the RLI, this would give rise to an impression that the target object alternately moves up and down, orthogonal to the direction of motion. In the circular version, this same effect would cause the moving segment to appear alternately nearer or further from the center of rotation. This apparent orthogonal shift occurring as the target passes in between the background junctions, combined with the apparent tilt effect at the junctions, would seem to nicely account for the connected “rocking” of the moving target experienced with the RLI.

Returning to the main theme of the current paper, the most obvious question is what causes the ITE? We suggest two critical factors. The first is the contrast step between the target and the tube. When this step is large, targets appear to be thinner than the control items, suggesting some sort of contrast sharpening. When the step is small, the targets appear fatter than control items, suggestive of assimilation (Nedimović & Zdravković, 2021). As expected, the global background also modulates the perceived step between target and tube contrast (see the online demo and Movie 3).

**Movie 3.** The effect of background contrast on the inner tube effect (ITE). The white and black rings are constant, but their relative sizes appear to change as the background modulates. Press any key to freeze the animation. https://maltacogsci.org/ITE/ITE_Movie3.html

The second critical factor appears to be the narrow “tube-like” border of the inducing surround. As can be confirmed in the online demo, increasing the width of the tube greatly weakens the apparent size discrepancy between the illusory targets and the control items (see also Figure 2C). The contour edges of both tube and target are thus clearly important, suggesting a link to some of the “phenomenal phenomena” reported by Gregory and Heard (1983).

Although we currently have no definitive explanation for the mechanisms behind the ITE, we believe the effect is compelling enough to warrant further study. We hope that by describing the effect in the context of the RLI, others may be encouraged to join us in trying to find a solution.

## References

[bibr1-20416695241249129] Gregory R. L. Heard P. F. (1983). Visual dissociations of movement, position, and stereo depth: Some phenomenal phenomena. The Quarterly Journal of Experimental Psychology, 35(1), 217–237. 10.1080/14640748308402127 6681185

[bibr2-20416695241249129] Nedimović P. Zdravković S. (2021). Lightness contrast & assimilation: Testing the hypotheses. Primenjena psihologija, 14(3), 253–275. 10.19090/pp.2021.3.253-275

[bibr3-20416695241249129] Thornton I. M. Todorović D. (2023). The rocking line illusion. I-Perception, 14(3), 1–5. 10.1177/20416695231184388. PMC10331093 37435314

[bibr4-20416695241249129] Todorović D. (2021). Polarity-dependent orientation illusions: Review, model, and simulations. Vision Research, 189, 54–80. 10.1016/j.visres.2021.09.003 34628261

